# Small object detection method with shallow feature fusion network for chip surface defect detection

**DOI:** 10.1038/s41598-022-07654-x

**Published:** 2022-03-10

**Authors:** Haixin Huang, Xueduo Tang, Feng Wen, Xin Jin

**Affiliations:** 1 School of Automation and Electrical Engineering, Shenyang Ligong University, Shenyang, 110159 China; 2 School of Information Science and Engineering, Shenyang Ligong University, Shenyang, 110159 China

**Keywords:** Electrical and electronic engineering, Mechanical engineering

## Abstract

The development of intelligent manufacturing often focuses on production flexibility, customization and quality control, which are crucial for chip manufacturing. Specifically, defect detection and classification are important for manufacturing processes in the semiconductor and electronics industries. The intelligent detection methods of chip defects are still challenge and have always been a particular concern of chip processing manufactures in an automated industrial production line. YOLOv4 method has been widely used for object detection due to its accuracy and speed. However, there are still difficulties and challenges in the detection for small targets, especially defects on chip surface. This study proposed a small object detection method based on YOLOv4 for small object in order to improve the performance of detection. It includes expanding feature fusion of shallow features; using k-means++ clustering to optimize the number and size of anchor box; and removing redundant YOLO head network branches to increase detection efficiency. The results of experiments reflect that SO-YOLO is superior to the original YOLOv4, YOLOv5s, and YOLOv5l models in terms of the number of parameters, classification and detection accuracy.

## Introduction

### Problem description and motivation

The development of intelligent manufacturing often focuses on production flexibility, customization and quality control, which are crucial for chip manufacturing. Specifically, defect detection and classification are important for manufacturing processes in the semiconductor and electronics industries^1^. Defect detection is critical for efficient product quality control. The traditional detection method relies on labor, and long-term manual detection can cause low detection efficiency and high rate of missing inspections. In addition, most of the defective chips are either recycled or reprocessed, and those cannot be reprocessed often get scrapped directly^2^.

Thus, it is also necessary to classify defective chips into types of defection for more effective processing. It provides rich information for production faulty inspection. However, most of the detection methods are still based on manual detection methods^3^. Therefore, the intelligent detection methods of chip defects are still challenge and have always been a particular concern of chip processing manufactures in an automated industrial production line^2^.

**Figure 1:**
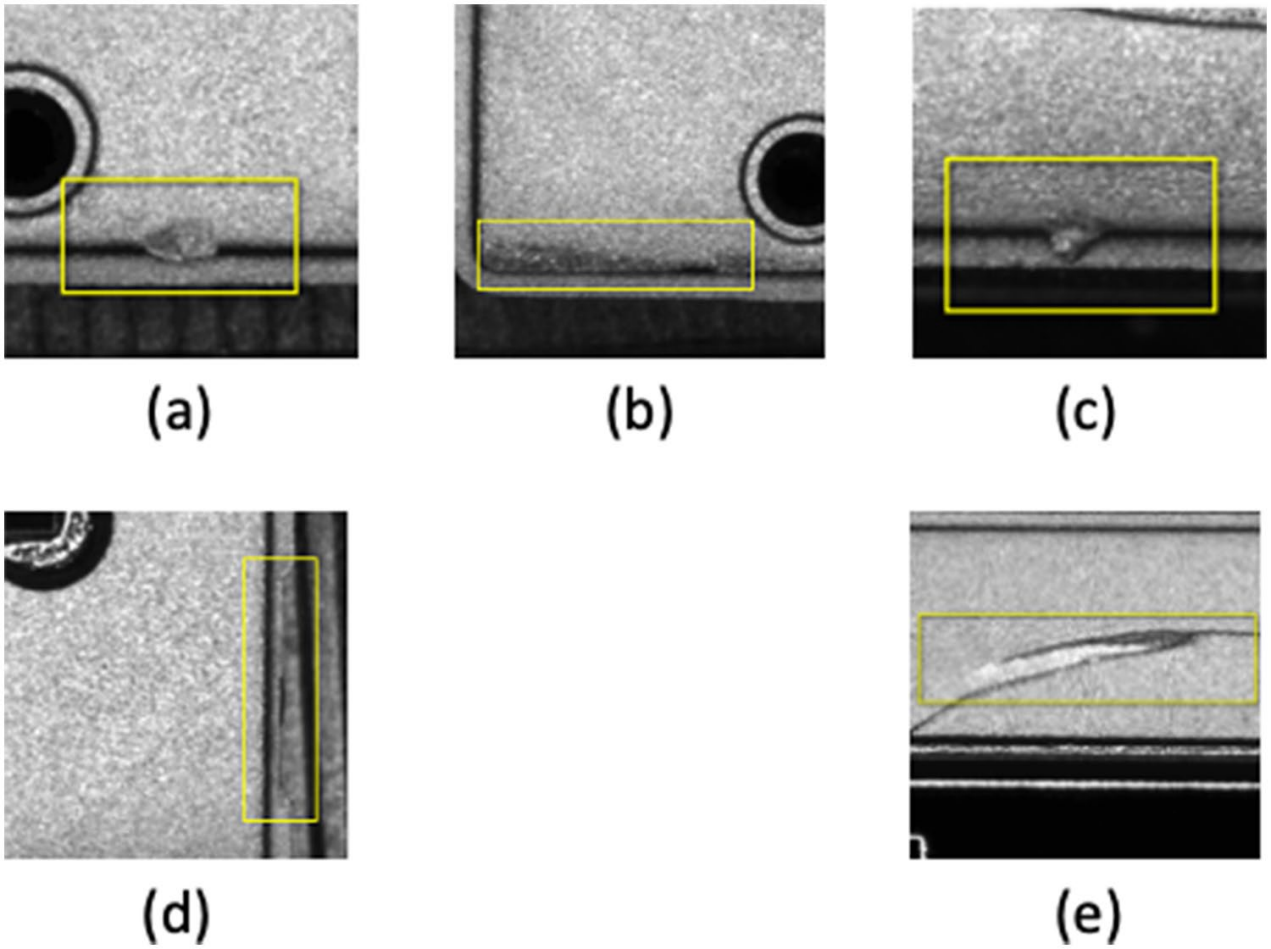
Common chip surface defects, (**a**) large area missing, (**b**) small area missing, (**c**) excess solder, (**d**) pits, (**e**) breach. (**c**) excess solder characterized by a bulge at the margin of chip, with a very small thickness of about 0.1 mm. (**d**) pit defect characterized by an irregular depression. (**e**) breach defect characterized by a width of 0.3mm and a length of 1mm.

There are various types of chip defects such as interfacial stress and solder joint reliability of the chip packages could occur during chip manufacturing process^4^. This study focuses on defects that related to large area missing at the edge, small area missing at the edge, excess solder and pits, and breach defect, see Fig. 1. For example, Fig. 1a,b are considered to be the same kind of defect but with different dimensions.

**Figure 2:**
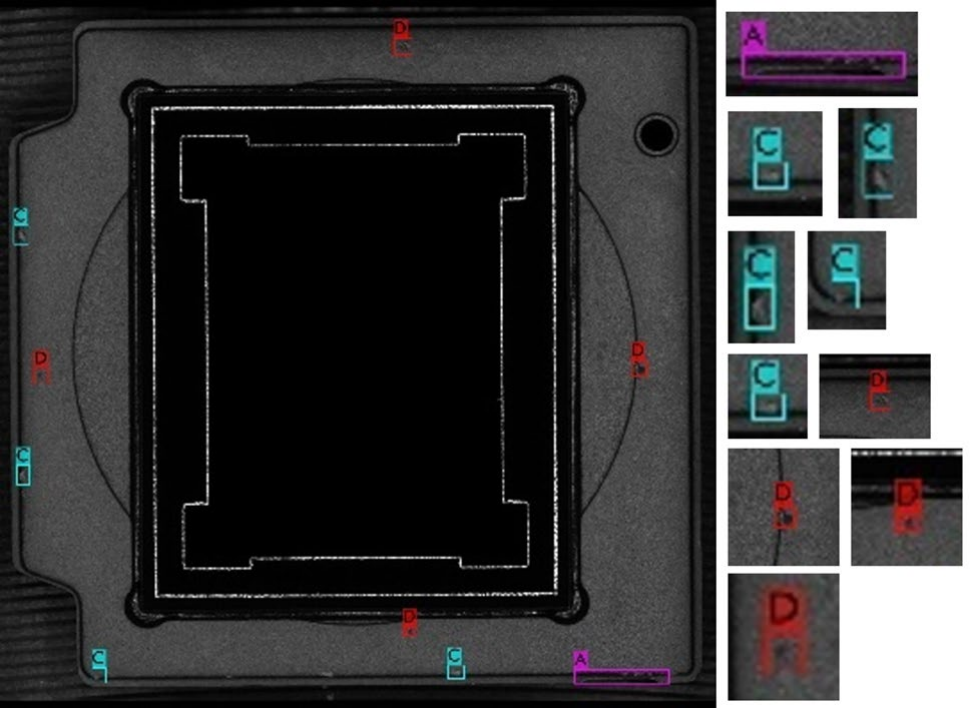
Result of SO-YOLO defect detection on sample chip image.

You Only Look Once v4 (YOLOv4) method has been widely used for object detection due to its accuracy and speed. However, there are still difficulties and challenges in the detection for small targets, especially the defects on chip surface. Therefore, this study proposed a small object detection method (SO-YOLO) based on YOLOv4 for small object in order to improve the performance of detection. For example, the result of the SO-YOLO small object detection is shown in Fig. 2.

### Contributions of this study

The proposed architecture of SO-YOLO is inspired by YOLOv4 network model and three modifications were made specifically for small object detection.

First, a new Feature Fusion method that expands the fusion of shallow features is proposed to satisfy small object detection and further improve the network prediction accuracy without reducing the speed. In order to improve the accuracy of small object detection, we made modifications for the Feature Fusion (FF) module, and network pruning. Instead of a normal Feature Fusion, the SO-YOLO adapts module based on Convolution Neural Networks (CNNs). Deep CNNs can learn hierarchical features in different layers which capture information from different scale objects. Specifically, spatial-rich features in shallow layers have higher resolutions and are more beneficial to the detection of small objects^5,6^. However, DSSD and FPN^7^ leverage the deconvolution layer from the top-most feature maps which may lost the majority of fine details for small objects. And these systems based on fusion features carry out connections for every prediction layer, which means more additional layers result in more computational cost at the same time. Increasing the number of fusion layers can better retaining the information of the targeted object^8^. Therefore, it is difficult to accurately detect small object relying on top-level layers only. The proposed Feature Fusion method is more appropriate for small object detection.

Second, the k-means++ clustering algorithm is used to optimize the number and size of anchor box adaptively during training. In order to improve the performance of defect detection on small object, it is necessary to have priori boxes adapted to the sample size first. Then the prior anchor boxes are used for detection, which can enhance the adaptability of the prediction scale.

Third, for the purpose of small object detection, redundant YOLO head network branches are removed except the large-scale feature layer, in order to reduce the model parameters and calculation. Due to the size of chips is approximately 6.6 $\times$ 6.6 millimeters (mm), and the average defect area size is approximately 0.3 $\times$ 0.3(mm), the common detection methods are not appropriate for small object detection^9^. Therefore, all network branches are removed except the large-scale feature layer.

## Related work

In recent years, machine learning methods has been widely used in surface defect detection and quality control^8,10^. For instance, detector based on RCNN family (R-CNN^11^, Fast R-CNN^12^, Faster R-CNN^13^, and Mask R-CNN^14^) algorithm converts the defect detection problem into a two-stage object detection problem^15^. It outperforms numerous algorithms in terms of detection accuracy. However, this kind of approach requires longer computational processing time comparing to one-stage detector such as single-shot multibox detector (SSD)^16^ and YOLO^5^. Especially, the YOLOv4^6^ algorithm has fast-operating speed and optimized parallel calculations for object detection.

More accurate detectors such as Fast R-CNN and Faster R-CNN are proposed by RGB et al recently^11,12^. Especially, the Faster R-CNN which inspired by Spatial Pyramid Pooling Net (SPPNet)^17^, putting forward the Region Proposal Network (RPN). An RPN is a fully convolutional network that simultaneously predicts object bounds and object scores at each position. Nevertheless, the top-most feature maps conflict with objects at different scales in images due to their fixed receptive field^18^. There is little in-formation left on the top-most features especially for small objects, so it is hard to be used on our study about chip surface defects detection.

Liu et al. proposed a method named SSD, which predicted objects by using multi-scale feature maps. SSD used the features from the shallow layers to detect smaller objects, while exploited the features from the deeper layers for bigger objects detection. However, due to lack of deeper semantic features, this method has a poor detection effect on small objects. Afterwards, Fu et al^19^. proposed a deconvolutional single-shot detector (DSSD), which increased lots of context information with using deconvolution layer. Alt-hough, DSSD algorithm realized a better detection accuracy, cost more prediction time. Deep-learning methods began being applied more often to surface-defect classification and detection problems shortly after the introduction of AlexNet^20^. Domen Tabernik et al.^21^ proposed a two-stage approach with segmentation network and the decision network, for the surface-quality control. The approach is suited to learn from a small number of detected training samples, but can still achieve state-of-the-art results. The work by Lv, et al.^22^ showed that for deep metallic surface defect detection the limited data scale and defect categories causing existing defect datasets are unavailable. To address this problem, they proposed an end-to-end defect detection network (EDDN), which based on the Single Shot MultiBox Detector. L, Xu et al.^23^ proposed a weakly supervised detection framework which uses localization and decision networks to predict the location and probability of defects simultaneously on a small subsets of defect samples and developed a new loss function. Experimental results exhibited a 99.5% recognition accuracy. Li, Y et al.^9^ proposed a detection method for surface defects, which combines the SSD network with the base convolution neural network MobileNet. In the pre-processing phase, they presented a regional planning method to cut out the main body of the defect, which reduces the redundant parameters and improve detection speed and accuracy. Liu et al.^8^ directly adopted the YOLOv3 as the meta structure for small object detection on Unmanned Aerial Vehicle Perspective, but they optimized the Resblock in darknet by concatenating two ResNet units that have the same width and height. Experimental results show a distinct performance improvement. Xu et al.^3^ proposed a small data-driven CNN for roller subtle defect inspection via an ensemble method for small data preprocessing. SDD-CNN applied LD to solve the imbalance in class distribution and presented the SSDA method to extent dataset, which had a good performance in the defect classification.

In today’s industrial product inspection field, automated inspection techniques such as deep learning and machine learning are gradually replacing traditional manual-based inspection methods^15,24^. Real-time detection has become the goal pursued by industrial product defect detection. Although deep learning has achieved better results in industrial defect detection, it still faces challenges such as incomplete defect data sets, small samples, and small targets.

## SO-YOLO method

### Detection algorithm framework

The common object detection framework consists of the backbone, neck, and head networks. The backbone network, such as the VGG Net, ResNet^7^, Inception Net^25^, is used for extracting features from the image. The neck network, such as Feature Pyramid Network (FPN)^26^, Path Aggregation Network (PANet)^27^, BiFPN^28^, has been widely used for merging the features of each layer from the bottom-up pathway or the top-down pathway. With the information extracted and processed from the backbone and the neck networks, the head network can be used for prediction. The head network is often categorized into one-stage object detector such as YOLO and SSD, and two-stage object detector such as the R-CNN series.

### SO-YOLO network structure

**Figure 3:**
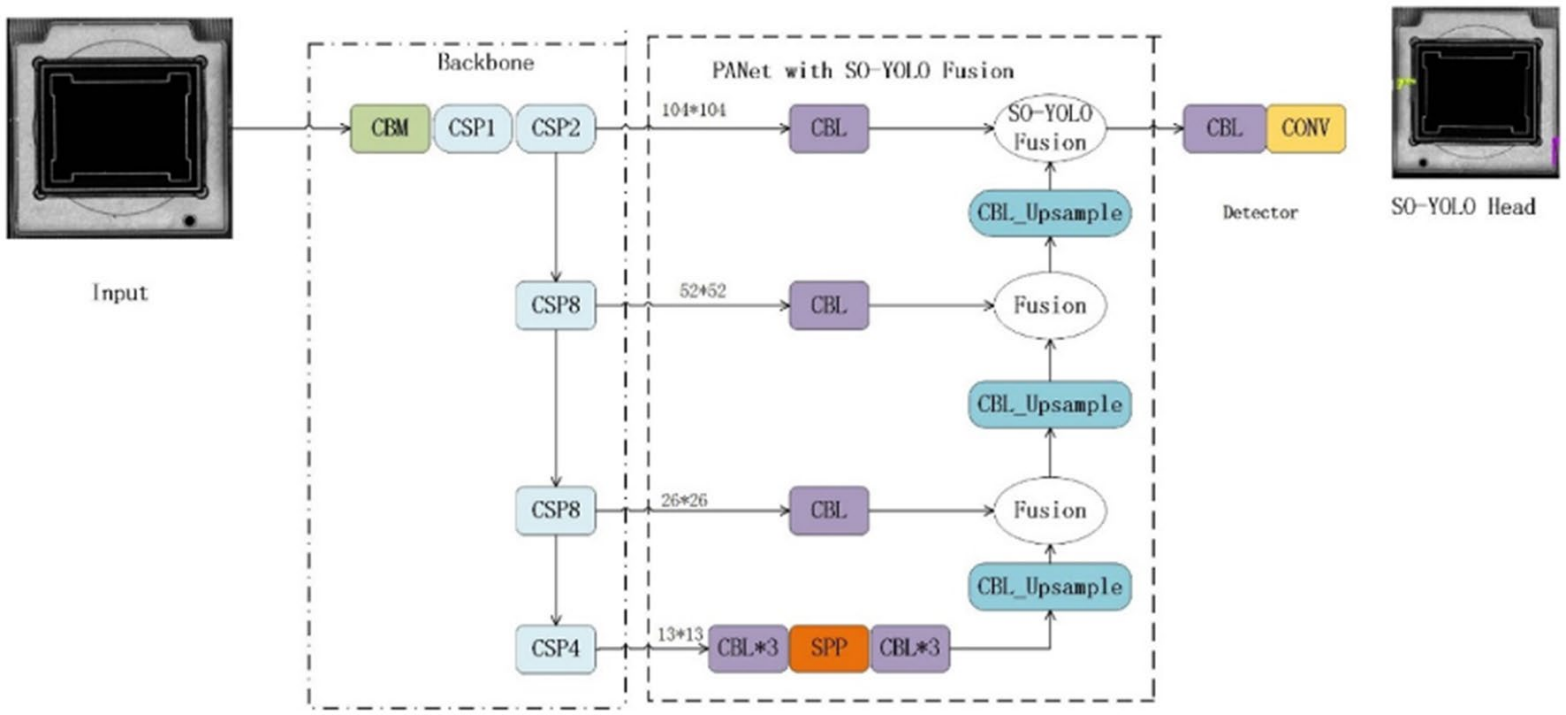
The architecture of the proposed SO-YOLO.

The SO-YOLO network structure consists of four modules, which are the input model, backbone, modified PANet and a detector network, see Fig. 3. The main difference between YOLOv4 and SO-YOLO is the structure of PANet.

Since lack of shallow features extraction can cause small object miss-detection and lower the detection accuracy, PANnet is modified in SO-YOLO to reserve shallow feature information. This modification involves two stages, see Fig. 3. First, input images features are extracted by the backbone network of SO-YOLO. Second, it regresses multiple bounding boxes in the image, and then classifies the objects in each bounding box with a convolutional neural network. PANet with SO-YOLO fusion is proposed to increase both precision and speed of chip defects detection. Lastly, the detector network is used to detect chip defects. PANet is a two-way top-down and bottom-up fusion backbone network, and a bottom-up network is added on the basis of FPN, which is a supplement to the shallow location information in FPN fusion results.

#### Feature fusion method for small objects

Handling feature scale issues is crucial for small object detection. Specifically, deep semantic-rich features can strengthen shallow spatially-rich features. Feature fusion, which is the integration of multiple different feature information, can obtain both deep se-mantic-rich features and shallow spatially-rich features. So, the method of feature fusion is particularly important for this study.

**Figure 4:**
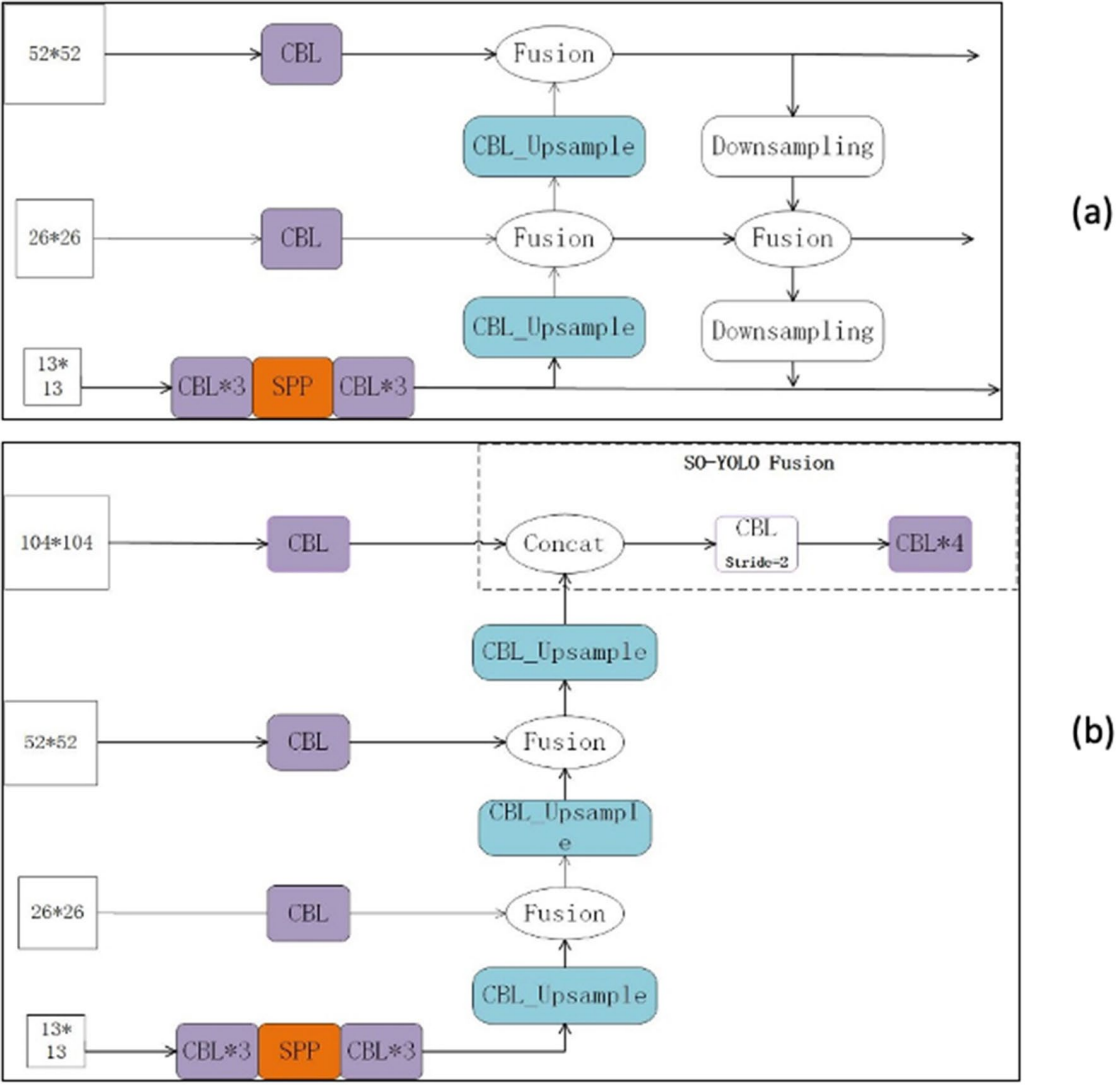
(**a**) Original fusion structure in YOLOv4 and (**b**)Modified fusion network in SO-YOLO.

The PANet structure of YOLOv4 is shown in Fig. 4a. For small objects detection with YOLOv4, first, it up-samples the 13$\times$13 feature map obtained from SPP network to 26$\times$26, and follow with a fusion between the feature maps. Then, it up-samples the 26$\times$26 feature map to 104$\times$104 directly. Lastly, it contacts the two feature layers 26$\times$26 and 104$\times$104 respectively.

The PANet of YOLOv4 contains both of the shallow and high-level information. However, for small object detection it might have some disadvantages. For instance, up-sampling 26$\times$26 straight up to 104$\times$104 may lose information for prediction. The number of channels reduced from 256 to 128 may lose information as well. Moreover, the scale of 104$\times$104 may increases the amount of model calculation for head network. The Feature Fusion network of proposed SO-YOLO is shown in Fig. 4b.

The structure of modified PANet is shown in Fig. 4b. Instead of up-sampling 26$\times$26 to 104$\times$104 directly, there is a feature fusion after SPP for each of the feature maps of 26$\times$26 with 512 channels, 52$\times$52 with 128 channels, and 104$\times$104 with 128 channels respectively. Compare to YOLOv4 the proposed SO-YOLO Feature Fusion network added a fusion layer, see Fig. 4.

#### The pruning network

**Figure 5:**
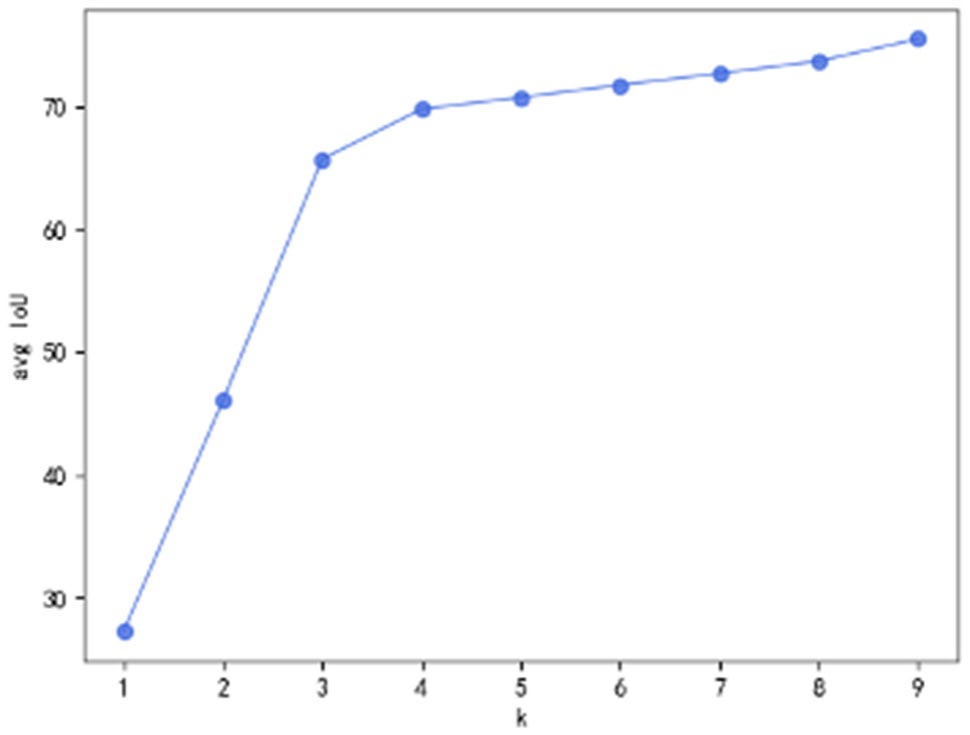
Average IOU results on different number of anchor boxes.

Enhanced Prior anchor Box. K-means++ clustering algorithm is used to estimate the number and aspect ratio of prior anchor boxes such as the amount and size of the boxes. Then the prior anchor boxes can enhance the ability for determining bounding boxes. The interval of K was set as 1 to 9. As shown in Fig. 5, the results implies that 3 is a knee point of K, which cause a reasonable avg IoU and model complexity. Therefore, the number of cluster centers is determined to be 3, and the size of enhanced bounding boxes obtained on this basis are (11, 11), (10, 51), (56, 10) respectively.

SO-YOLO Head. The original YOLOv4 algorithm allocates 3 bounding boxes for each of the three feature maps with scales 52$\times$52, 26$\times$26, and 13$\times$13 for prediction. For SO-YOLO, deep layer features such as 26$\times$26 and 13$\times$13 with semantic-rich information have larger receptive fields, which are suitable for detecting small objects specifically. Studies showed that limited receptive filed has difficulty in small object detection^8^. Hence, this study discarded the small-scale feature maps and reserved the 52$\times$52 feature map, which also reduced the complexity of network.

## Experiments and results

### Chips-surface defect images dataset

Due to lack of publicly available defective chip dataset, this study collected images from a domestic factory. Totally 896 images obtained by two industrial cameras. The dataset images used in this experiment are of high pixel size (2081*2127). The percentage of defective objects in a single image is only 0.36% at minimum. According to the definition of the International Society for Optics and Photonics (SPIE), the object of this study belongs to the field of small object research. Images were divided into training and test datasets with ratio of 9:1. The hardware configuration include one NVIDIA GeForce GTX1660 SUPER graphic card, AMD Ryzen 5 3500X 6-Core Processor, and 16GB RAM. The learning rate, attenuation coefficient and iteration were set to 0.001, 0.0005 and 10,000 times respectively. The darknet framework and Python 3.8 were used.

### Image preprocess

**Figure 6:**
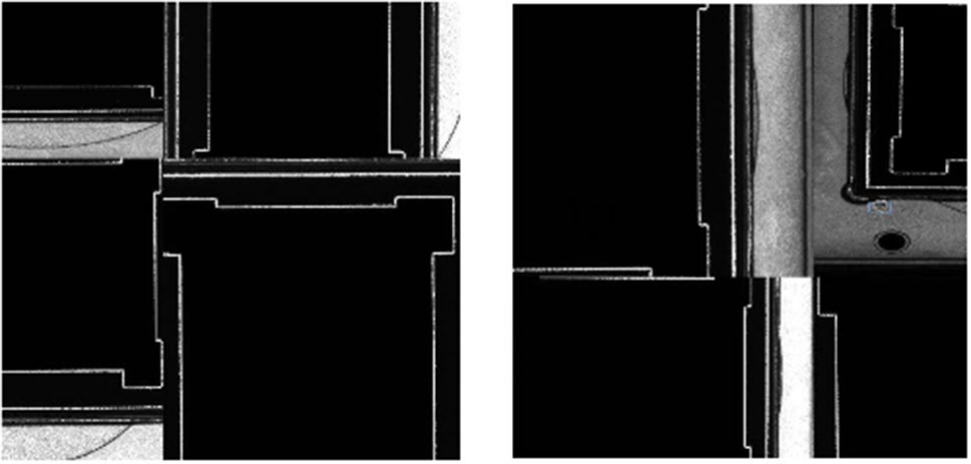
Demonstration of mosaic data enhancement method on sample image.

The original dataset was expanded using data augmentation methods, which include Geometric Transformation and Mosaic methods. Mosaic data augmentation is based on Cut-Mix theory. The difference is that there are two images spliced in Cut-Mix, but the Mosaic used four. The advantage of using Mosaic is that it enriches the background of image datasets. First, the dataset was expanded to 8 times of the original size using mirroring and rotation. Then, Mosaic method was used to merge images, see Fig. 6.

### Experimental results

Object detection performance is evaluated with Average Precision (AP) and mean Average Precision (mAP) between ground truth and predicted bounding box (IOU). To verify the result of the optimized Feature Fusion network, two experiments are performed in this study. The experiment results are shown in Table 1.

**Table 1:** Comparison of mAP and IOU on YOLOv4 and SO-YOLO. i.e., “2/128” means Resblock_body repeat 2 times and the dimension of feature map is 128.

Method	Precision	Recall	F1 score	Average IOU%	mAP%
SO-YOLO-2/128	0.79	0.82	0.81	54.09	83.20
SO-YOLO-4/256	0.78	0.76	0.77	53.27	79.19
SO-YOLO-2/256	0.80	0.83	0.82	54.30	86.00
YOLOv4	0.79	0.82	0.80	54.38	82.59

Experiment results reported in Table 1 is the average of multiple experiments. It can be seen that increasing the dimension of feature map improved prediction precision. Comparing with SO-YOLO-2/128, which has the same setting of Resblock_body and feature map dimension as YOLOv4, model SO-YOLO-2/256 has outstanding performance. As shown in Table 1, mAP is increased from 83.20 percentage to 86 percentage. Due to the compromise selection of the prior anchor box, the IOU of SO-YOLO is lower than YOLOv4. Comparing to YOLOv4 the proposed method has better performance and also well-balanced accuracy and processing time, see Table 2.

**Table 2:** Comparison of network model parameters.

Version	BFLOPS	Num of layers
SO-YOLO-2/128	48.775	149
SO-YOLO-4/256	55.398	154
SO-YOLO-2/256	53.624	148
YOLOv4	75.843	161

**Figure 7:**
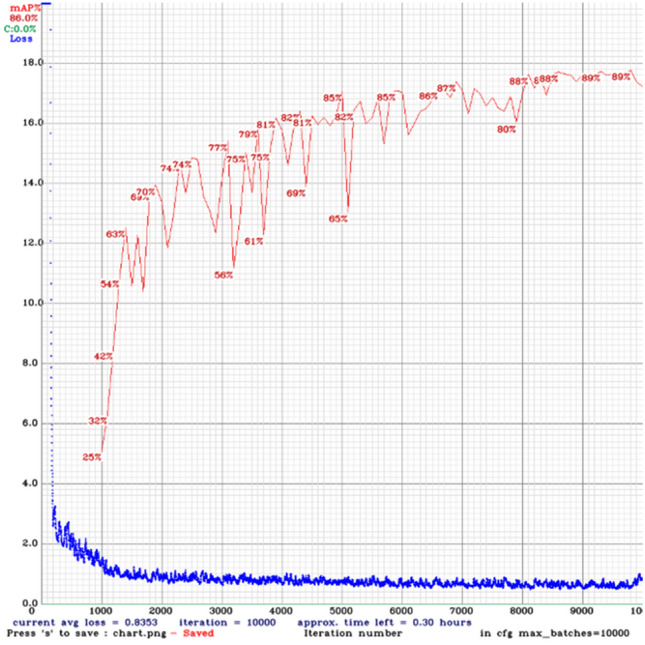
Training loss and mAP of SO-YOLO-2/256.

Table 2 showed the SO-YOLO-4/256 has the greatest number of layers and BFLOPS, but its performance is the worst. The reason could be increased number of parameters and unbalance of semantic and spatial information. Comparing with YOLOv4, the number of network layers of SO-YOLO-2/256 reduced from 161 to 148, and the model parameters reduced from 75.843BFLOPS to 53.624BFLOPS. And it can be viewed in Fig. 7 that the SO-YOLO algorithm has converged after iterating to 8000 times.

In order to evaluate the performance of SO-YOLO model, experiments were conducted to compare it with YOLOv4, YOLOv5s, and YOLOv5l, see Table 3. This experiment used same training data set on these models. The results showed that the SO-YOLO model had better performance comparing to YOLOv4, YOLOv5s and YOLOv5l. First, SO-YOLO had the highest mAP. Second, SO-YOLO and YOLOv5s have very close F1 scores, but the accuracy of the YOLOv5s model is lower than the SO-YOLO model. Although the complexity of the YOLOv5s model is simpler. Third, YOLOv5l is the weakest in terms of performance in this comparison. Hence, this experiment confirmed the importance of network depth and shallow information for small object detection. In conclusion, our model not only reduces the complexity of the model, but also can improve the small object detection accuracy.

**Table 3:** Comparison of detection accuracy between SO-YOLO and YOLOv4, YOLOv5s, YOLOv5l.

Model	F1	mAP	BFLOPS
YOLOv4	0.80	0.826	75.843
YOLOv5s	0.84	0.821	14.0
YOLOv5l	0.73	0.711	123.1
SO-YOLO	0.84	0.86	53.624

## Discussion

In order to improve the classification and defects detection on chip surface, SO-YOLO is proposed in this study. This study adopted the CspDarknet53 as the meta network structure, and optimized entire PANet using a new method of Feature Fusion. As a result, this method can enhance the receptive field by choosing appropriate layers to make a fusion. Moreover, this study also adopted k-means++ as prior anchor estimation method. Lastly, Mosaic data augmentation method was used to preprocess the dataset. The results of experiments reflect that SO-YOLO is superior to the original YOLOv4 model in terms of the number of parameters, classification and detection accuracy. The proposed feature fusion method and pruning model may be useful for small object detection. We hope detection accuracy and reducing model complexity can be improved in the future study.
